# Fractal model and Lattice Boltzmann Method for Characterization of Non-Darcy Flow in Rough Fractures

**DOI:** 10.1038/srep41380

**Published:** 2017-02-01

**Authors:** Yang Ju, Qingang Zhang, Jiangtao Zheng, Chun Chang, Heping Xie

**Affiliations:** 1 State Key Laboratory of Coal Resources & Safe Mining, China University of Mining & Technology, Beijing 100083, China; 2 State Key Laboratory for Geomechanics & Deep Underground Engineering, China University of Mining & Technology, Xuzhou 221116, China; 3 School of Mechanics and Civil Engineering, China University of Mining and Technology, Beijing 100083, China; 4 Department of Engineering Mechanics, School of Aerospace, Tsinghua University, Beijing 100084, China; 5 Key Laboratory of Energy Engineering Safety and Mechanics on Disasters, The Ministry of Education, Sichuan University, Chengdu 610065, China

## Abstract

The irregular morphology of single rock fracture significantly influences subsurface fluid flow and gives rise to a complex and unsteady flow state that typically cannot be appropriately described using simple laws. Yet the fluid flow in rough fractures of underground rock is poorly understood. Here we present a numerical method and experimental measurements to probe the effect of fracture roughness on the properties of fluid flow in fractured rock. We develop a series of fracture models with various degrees of roughness characterized by fractal dimensions that are based on the Weierstrass–Mandelbrot fractal function. The Lattice Boltzmann Method (LBM), a discrete numerical algorithm, is employed for characterizing the complex unsteady non-Darcy flow through the single rough fractures and validated by experimental observations under the same conditions. Comparison indicates that the LBM effectively characterizes the unsteady non-Darcy flow in single rough fractures. Our LBM model predicts experimental measurements of unsteady fluid flow through single rough fractures with great satisfactory, but significant deviation is obtained from the conventional cubic law, showing the superiority of LBM models of single rough fractures.

Natural rocks are generally composed of complex and heterogeneous fractures, which provide storage capacity and migration paths for oil, gas and water resources^1^,^2^. Irregular morphology of rock fracture significantly complicates the fluid flow, resulting in unpredictable engineering processes for enhancing geothermal-reservoir mining, geological sequestration of carbon dioxide and groundwater remediation, etc. The irregular morphology of single rock fracture significantly influences subsurface fluid flow and gives rise to a complex and unsteady state that typically cannot be appropriately characterized using simple laws^3^,^4^,^5^,^6^. In addition, coal-mine water-bursting disasters, coal-gas outburst accidents, dam disasters and rock-slope failures have shown to be closely related to fluid seepage, the dynamic evolution of rock fractures and coupled stress–fluid flow processes^7^,^8^. A clear and detailed knowledge on the fluid flow and its interaction with stress in fractured media is critical when addressing the above engineering issues.

Being the basic element of the complex fracture network, a single fracture with its morphology controls the fluid flow initiation and development in the network. Some mechanical model approaches have been proposed to investigate the properties of fluid flows through single fracture and fracture networks, such as representative elementary volume (REV), discrete fracture network (DFN), hydrological-mechanical-chemical (HMC), thermos-hydro- mechanical (THM) approaches, and parallel plate and channel models^1^,^9^,^10^,^11^,^12^,^13^,^14^,^15^,^16^,^17^,^18^,^19^,^20^,^21^. Liu *et al*.^1^ proposed the hydrological-mechanical-chemical (HMC) method to explain the enigmatically spontaneous changes in permeability that develop within single fracture in limestone under *in-situ* conditions. The parallel-plate model, which considers contact areas between matrix and fluid and artificial fractures, has been proposed to evaluate the effects of contact area and surface roughness on fluid flow in rock fractures^22^. To adapt and simulate the fluid flow in the dominant passageway, Tsang *et al*.^19^,^20^ presented a channel model for fluid flow through a tight fracture subjected to high stresses. However, the morphology of the contacted surfaces that stresses applied appeared to be so irregular that accurate definition on the structures of channel walls and the properties of fluid flow using mathematical or physics tools became extremely complicated.

The fractal-dimension method (FDM) provides an effective way to accurately describe the fracture morphology, comparing traditional methods including the bump-height^23^ and the joint roughness coefficient (JRC)^24^,^25^. Barton *et al*.^25^ provided a revised method from a coupled joint behaviour model using the joint roughness coefficient (JRC) and verified that the properties of seepage flow were dominated by the morphology and connectivity of the passageway formed by the untouched fracture surface. However, the bump-height method of measuring every point’s bump-height in rock fracture is extremely difficult to apply in engineering practices, and the JRC method only qualitatively characters the ten known fracture types. By contrast, the fractal-dimension method can be used to quantitatively describe the fracture morphology with a much wider application. To the best of our knowledge, very few numerical studies have applied the fractal dimension method to estimate the effect of irregular fracture surface on the permeability and fluid velocity field.

The common cubic law (CCL), based on the smooth parallel-plate assumption that the aperture changes can result in a change of conductivity as much as three orders of magnitude at moderate compressive stress levels, has been widely applied to the analysis of seepage-flow behaviours in rock masses. The CCL has also been used to investigate the properties of fluid flow and the mechanisms of hydraulic-mechanical coupling in fractured rock^1^,^9^,^10^,^11^,^12^,^13^,^16^. In CCL modelling, Darcy’s-law is implemented, which forms the basis of hydrogeology and is one of the most famous law that describes fluid flow through a porous medium^26^. However, substantially differing from Darcy’s law, fluid flow in natural rough fractures can be influenced by a range of factors^27^ including surface roughness, fluid-matrix interface area, aperture, connectivity^28^,^29^,^30^, unit width flux and hydraulic gradient. A comprehensive and detailed study on the fluid flow through natural rough fractures is needed.

Comparing to CCL of computational fluid dynamics (CFD), the Lattice Boltzmann Method (LBM) provides a powerful technique for modelling single/multiple phase flow in porous and fractured media with complex geometries^27^,^31^,^32^,^33^,^34^,^35^. It is a kinetic-based mesoscopic approach that bridges the micro- and macro-scale, offering distinctive advantages in simulation fidelity and computational efficiency^36^. The LBM has been widely applied to study fluid flow in porous media. Ju *et al*.^37^ presented the dynamic methane flow and distribution at microscale in porous sandstones subjected to force-induced deformation through LBM, and the method effectiveness in complex porous structure was validated by experimental observations. Pazdniakou and Adler^38^ and Gao *et al*.^39^ used the LBM to investigate the dynamic permeabilities of porous media and the multicomponent fluid-flow in complex porous media, respectively. Fan and Zheng^40^ studied the seepage flow in a complex and rough fracture network using LBM. However, limited to an effective tool to describe the fracture morphology, little work has been published on the effects of fracture roughness on flow properties in single fractured rock.

The main objective of this study is to investigate the fluid flow in single rough fractures and the effect of irregular morphology of fractured rock by combining fractal-dimension method and LBM. The fractal governing function was embed for generating single rough fracture models with fractal dimension (*D*) varying from 1.0 to 1.5 and LBM for fluid (water) flow. Modelling results were validated by experimental measurements under the same conditions. The accuracy and efficiency of this numerical method with considering the non-Darcy flow field were analyzed and discussed with respect to the velocity-field distributions and equivalent permeabilities in the fractured models.

## Results

### Velocity distribution field

**Figure 1:**
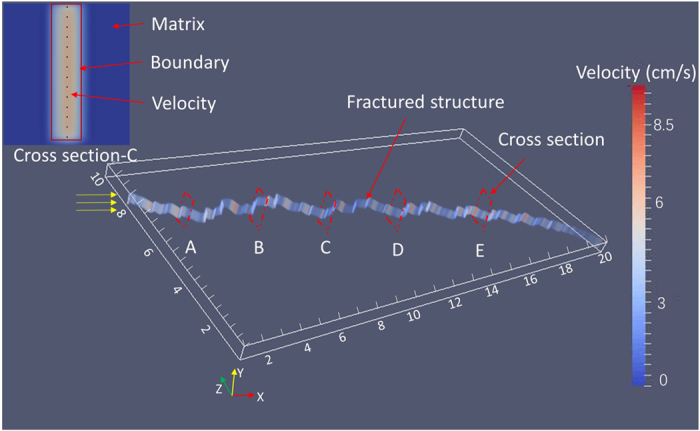
Schematic representation of water velocity distribution and the five cross sections selected in the single rough fracture model of *D* = 1.5. Water flows along x-axis direction (marked by the yellow arrows) perpendicular to y-z plane from the inlet (x = 0) to the outlet at x = 20 cm. The Inset image shows an example of the cross-section, in which the blue colour indicates matrix, the yellow indicates fracture, the red line indicates the model boundary and the 14 black dots indicate the symmetrically selected lattice points for further analysis on velocity distribution. The legend depicts the velocity magnitudes, in which blue indicates the minimum value and red represents the maximum number.

**Figure 2:**
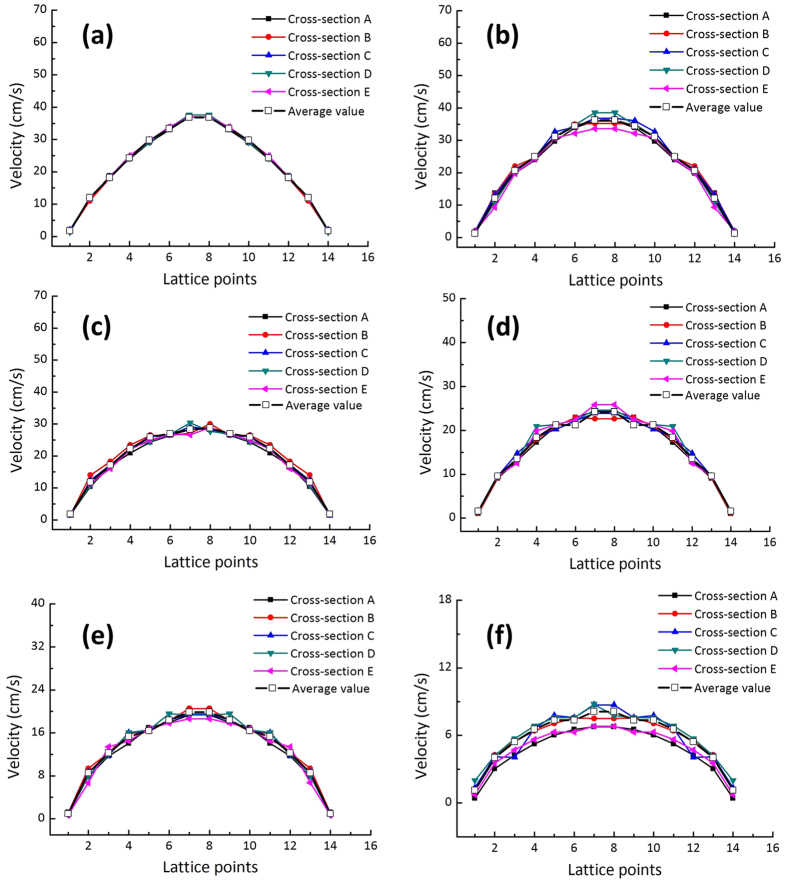
Velocity distribution of water flow along the 14 selected lattice points in the five selected cross sections of the single rough fracture models with varied fractal dimensions. From (**a**–**f**), the fractal dimension *D* is equal to 1.0, 1.1, 1.2, 1.3, 1.4 and 1.5, respectively.

Figure 1 illustrates the velocity distribution of water over the entire fracture space, with detailed structural information from the fractal model of *D* = 1.5. To further investigate the influence of surface roughness and quantify the modelling results by experimental measurement^7^, as shown in Fig. 1, we evenly selected five cross sections (A–E) along the flow pathway of a single rough fracture model with a fractal dimension varying from 1.0 to 1.5. Each cross section includes 4,000 lattice points (100 × 40), from which 14 representative points (marked by the black dots in Fig. 1) were symmetrically selected to display the velocity distribution across the section. Figure 2 shows the distribution of water velocity over the five cross sections in fracture models with varying fractal dimensions (*D* = 1.0 to 1.5). Higher velocities in the centre of each cross-section were observed, with a decreasing trend from the centre to the both ends. These results show that in a single fracture with a constant fractal dimension, the variation of water velocity at different locations is small (see Fig. 2), even if the local roughness of the fracture is different. In the smooth flat fracture (*D* = 1.0), the flow velocity remained unchanged in the five different cross-sections.

### Permeability of single rough fracture

Table 1 shows the permeabilities in fractures with varying fractal dimension as determined from the cubic law (Equation (3)) (*k*_*f*_), the experiment measurement (*k*_*e*_) and the LBM simulation (*k*_*0*_).

## Discussion

**Figure 3:**
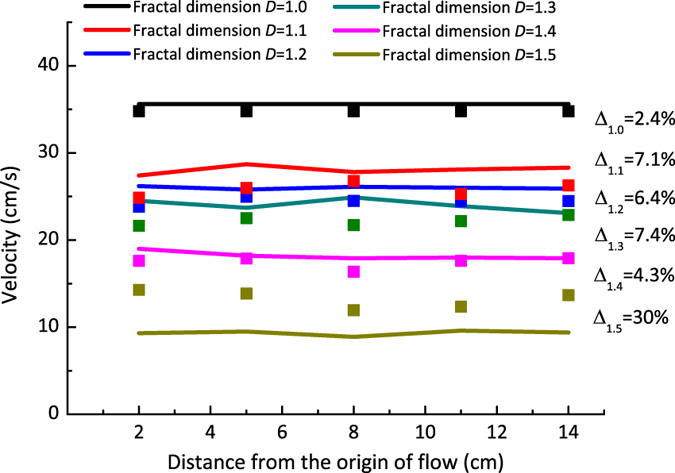
Experimental and simulated flow velocities over cross-sections of the stated fractal dimensions (*D*). The *D* values are provided in the figure. The lines represent modelling results and the symbols represent experimental data. ∆: average velocity deviation between the modelling and experiments for each fractal dimension.

Through the integral calculation of all the points over the entire cross section (y-z plane) of fracture with various fractal dimensions, the average velocity of water in different five cross-sections is obtained. Figure 3 illustrates the average velocity deviation between the LBM simulation and the experimental measurements over five cross-sections in various fracture models. The deviation between the numerical and experimental measurements is less than 10% for fractures with *D* = 1.0 to 1.4 (see Fig. 3). However, the deviation increases up to 30% for fracture with *D* = 1.5. The possible reason might be the discrepancy between the LBM numerical model of rock based on self-compiled programs and the physical cells of fractured rock. Nevertheless, from an engineering point of view, the case of deviation less than 10% would be acceptable, which will not significantly impact the general trend that the velocity evolves. The very rough fracture surface significantly influences the fluid velocity-field distribution. However, for a single fracture with constant fractal dimension, although the rough structure of the selected path segment was different, the average flow velocity did not change significantly, implying that the average flow velocity was independent of its local structural morphology.

**Figure 4:**
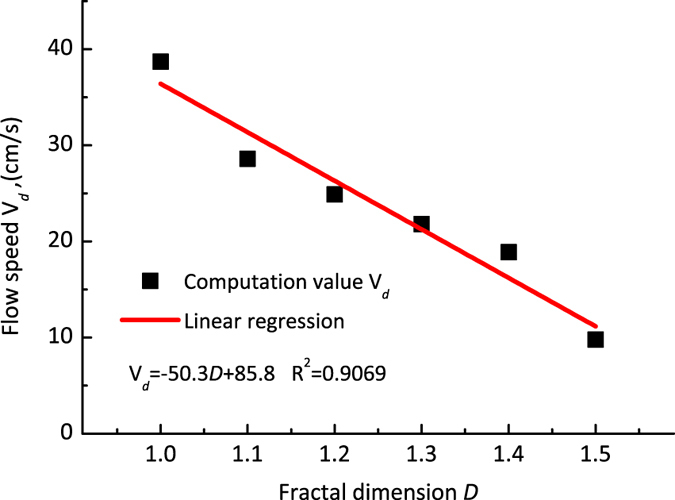
The linear regression between velocity-field distribution of water and the various fractal dimensions.

Figure 4 shows the linear correlation between the average water velocity over the entire fracture space in single rough fracture models, as simulated using the LBM. Furthermore, a comparison between the rough fractures with various fractal dimensions suggests that as the fractal dimension increases — that is, as the fracture roughness increases — the average velocity of the flow in any segment decreases, as does the mean velocity of the water flow through the entire path of the fracture.

The deviation between *k*_*0*_ and *k*_*f*_ increases significantly with fractal dimension and exceeds 30% when *D* = 1.5 (see Table 1). Meanwhile, it is noted that the deviation between *k*_*0*_ and *k*_*f*_ is less than 5% when *D* = 1.0. Same deviation trend applied to *k*_*e*_ and *k*_*f*_, but with much more significant increase in *D* = 1.5. The value keeps less than 15% as the fractal dimension is smaller than 1.4. The possible reason might result from the discrepancy between the LBM numerical model of rock based on self-compiled programs and the physical cells of fractured rock. In summary, it can be concluded that the permeability (*k*_*0*_) decreases with increasing in the fractal dimension. Meanwhile, the results for the non-Darcy flow obtained using the LBM approach deviated significantly from the results obtained from CCL, indicating its inconsistency and incapability for describing and representing the complex flow behaviours in the fractal models.

**Figure 5:**
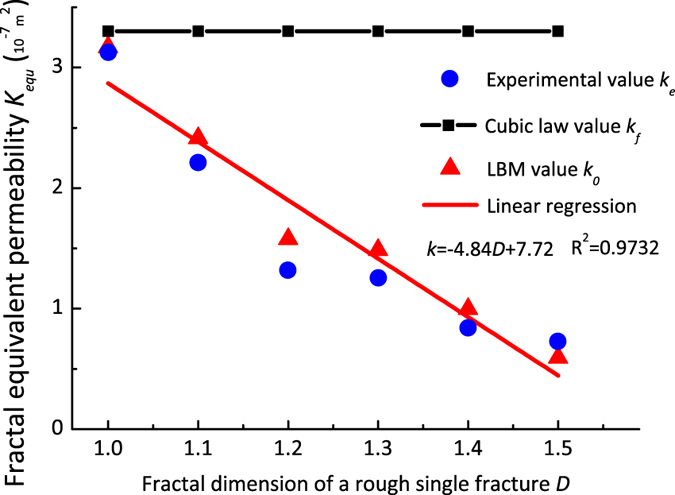
Variation in the equivalent-permeability coefficients (*k*_*f*_, *k*_*e*_, and *k*_*0*_) of water flows in fracture models with varying fractal dimensions.

The equivalent permeability coefficients (*k*_*f*_, *k*_*e*_ and *k*_*0*_) of water flow varying with the various fractal dimensions in the fracture models are plotted in Fig. 5. One can easily formulate the following linear relationship between the equivalent-permeability coefficients (*k*_*0*_) of a single fracture and the fractal dimension *D* of its rough fracture. These results suggest that the fractal equivalent permeability (*k*) decreases linearly as the fractal dimension of the rough structure (that is, roughness) increases, except for the case of the cubic law, where *k*_*f*_, is constant. We found that the LBM simulation results have a good consistency with the experimental measurement. Therefore, it seems to be an effective way to quantitatively characterize the spatial distribution of flow velocity, permeability, and the influence of the roughness on the fluid flow behaviour in the single rough fracture models with various fractal dimensions.

## Methods

### Fractures build up by FDM

A series of single rough fracture models with different fractal dimensions were constructed using the Weierstrass–Mandelbrot function, as implemented in self-programming functions. The Weierstrass–Mandelbrot function is formulated as refs 41, 42


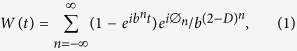


where *b* is a real number greater than 1, 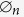
is any angle and D ∈ (1, 2) is the fractal dimension. The fractal governing function, *C(t*) is then the real part of *W(t*)^42^:


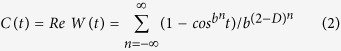


**Figure 6:**
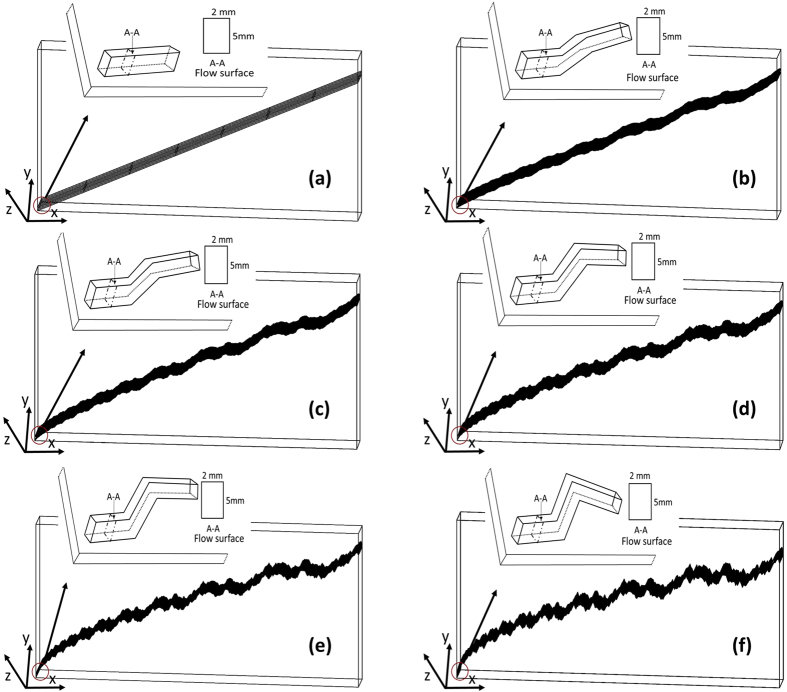
Single rough fracture models with varying fractal dimensions (1.0–1.5) representing different surface roughness, and the dimensions of flow surface that water flows through. *D* = 1.0 quantifies a smooth flat fracture, and the fracture roughness increases with *D* values. The fractal depth and width are 5 mm and 2 mm separately, and the total area of the flow surface (A-A) is 10 mm^2^.

Considering the flow surface (see Fig. 6) along the fracture depth, we implemented the Weierstrass–Mandelbrot function and the physical cells established in previous study^21^ to build up the single rough fracture models with various fractal dimensions. Figure 6 shows the single rough fracture models with various fractal dimensions, including the magnified inserts showing the detailed structure. The scale of the fractal model is 200 mm long, 100 mm wide and 5 mm thick in x, y, z axis, respectively. The scale and morphology of the fracture were kept identical to the cell used in the experiments. Further methodological details with experimental measurements and results can be found in Ju *et al*.^7^.

### Velocity field and permeability in single rough fractures

Permeability refers to the ability of a fluid flows through the fractured or porous rock. Permeability is typically given as a function of the fracture aperture, *b*_0_, under the conditions of isothermal and laminar flow between two parallel glass plates^31^:


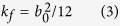


In this section, water velocity field and fracture permeability are investigated through LBM. Understanding the correlation between fracture morphology and permeability is thus important for accurately evaluating reservoir recovery and production rates. For that purpose, we adopted the distribution functions of flow velocity (equations (6, 7, 8, 9, 10, 11)) and the equation for permeability (equation (15)) to determine the permeability *k* of a fluid flowing through the single rough fracture. To simulate and analyse the effects of the rough surface on the fluid-flow behaviour in our models, the physical units including fluid pressure field *p*, macroscopic fracture aperture *L* and kinematic viscosity of fluid *v* were first transformed to the lattice units before determining the velocity-field distributions (Equations (12, 13, 14)). To enhance the accuracy of the simulation in the current context of fractured rocks and to reduce the computational time, we adopted the D3Q19 model^43^ to discretize the velocity at each lattice, and the single-relaxation-time Bhatnagar–Gross–Krook (BGK) approximation (equation (4)) was used to determine the movement and collision of fluid particles, which can be expressed as


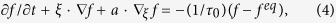


where *f(r, ξ, t*) refers to the velocity distribution, which is a function of the spatial position vector *r*, velocity vector ξ, and time *t*. By discretizing the left-hand terms of equation (4) in time and space and replacing the right-hand term of equation (4) by a first-order rectangle approximation, we can convert the equation to


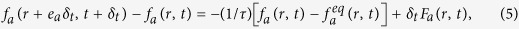


where *τ* = *τ*_0_/*δ*_*t*_ is the dimensionless relaxation time and *δ*_*t*_*F*_*a*_(*r, t*) refers to the external force term. In the D3Q19 model, the distribution function at the equilibrium state is defined as


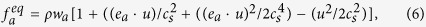



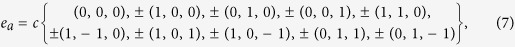



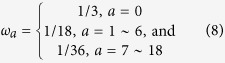



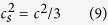


where *ω*_*a*_ is the weight coefficient, *c*_*s*_ is the sound velocity of a lattice and *c* is the lattice speed. *ρ* is the fluid density and *u* is the fluid velocity, which can be determined by equations (10) and (11):


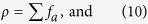



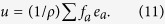


Before determining the velocity-field distributions in our LBM simulation, the macroscopic parameters of the physical units including fluid pressure field *p*, fracture aperture *L* and kinematic viscosity of fluid *v* were first transformed to the lattice units, which are determined by equations (12), (13) and (14):


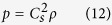



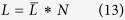



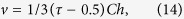


where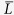 is the length of a lattice, *N* is the lattice number and *τ* is the relaxation time.

After obtaining *u*, the water permeability in different fracture models can be calculated by Darcy equation. Because the Reynolds number of the flow with the experimental measurement is lower than the critical value 2000, the viscous force prevails, indicating that the water flow in the rough fracture represents laminar flow^7^. Thus, assuming the fluid flow meets the condition of laminar flow in the representative micro-scale segment, the rock permeability can be determined as


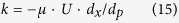


where *k* denotes the permeability of the medium, *x* refers to the direction of flow, −*d*_*p*_/*d*_*x*_ is the pressure gradient along the flow direction, *μ* is the water viscosity and *U* is the average velocity per unit area.

### Boundary and initial conditions

The single rough fracture models were generated with a gridding size of 2000 × 1000 × 50 pixels in the LBM modelling, representing 0.2 × 0.1 × 0.005 m in physical size. The relevant parameters and boundary conditions used in the numerical simulation were identical to the experimental set-up and as follows:

1. The density and viscosity of water is referred as 998.2 *kg/m*^3^ and 0.001003 *Pa* · *s*, respectively^7^. In order to make the simulation straightforward, we postulate the fluid flow within the fracture models as single phase flow.
2. The left boundary of the model was set as inlet, which was defined as a constant pressure boundary at 490 Pa. The right boundary of the model was set as outlet under atmospheric pressure (see Fig. 2). The initial velocity of the flow field was 0 m/s. The other parts of the model, with the exception of the fractal fracture, were set as ‘bounce-back boundaries’, indicating that the evolution of the particles was considered as head-on collisions of two particles.
3. Convergence: The simulation convergence was controlled by mesh generation, two- particle collision patterns, fluid property and iteration steps. The mesh resolution was set as 1 pixel to ensure convergence in the relatively small fractures. The modelling was stopped and the convergence results exported at iteration steps exceeded 8000 and the standard deviation of the average energy was less than 10^−4^.

### Experimental setups

To verify the effective of the LBM simulation, a series of single rough fracture physical cells with varying roughness were produced using the Weierstrass–Mandelbrot function and transparent polymethyl methacrylate (PMMA) material. A high-speed video camera was employed to record the fluid flow through the entire single rough fracture with a constant hydraulic pressure. The properties of fluid flow varying with the fracture roughness and the influences of the rough surface were analyzed. More details on the seepage experiments can be referred to Ju *et al*.^7^.

## Additional Information

**How to cite this article**: Ju, Y. *et al*. Fractal model and Lattice Boltzmann Method for Characterization of Non-Darcy Flow in Rough Fractures. *Sci. Rep.* **7**, 41380; doi: 10.1038/srep41380 (2017).

**Publisher's note:** Springer Nature remains neutral with regard to jurisdictional claims in published maps and institutional affiliations.

## Figures and Tables

**Table 1:** Permeabilities as determined from the cubic law (*k*_*f*_), the LBM simulations (*k*_*0*_) and the experimental measurements (*k*_*e*_)^7^.

Fractal *D*_*i*_	*k*_*0*_	*k*_*e*_	*k*_*f*_	Deviation	Deviation	Deviation
				|(*k*_0_ − *k*_*f*_)/*k*_0_|%	|(*k*_*e*_ − *k*_*f*_)/*k*_*e*_|%	|(*k*_0_ − *k*_*e*_)/*k*_*e*_|%
1	3.169	3.12	3.3	4.134	5.668	1.473
1.1	2.414	2.21	3.3	36.7	49.46	9.33
1.2	1.476	1.32	3.3	123.6	150.6	12.07
1.3	1.387	1.25	3.3	137.9	163.4	10.69
1.4	0.726	0.84	3.3	353.5	293.3	13.47
1.5	0.494	0.73	3.3	568.1	354.5	31.96

Note: the units of *k*_*0*_, *k*_*e*_ and *k*_*f*_ are 10^−7^ m^2^.
